# Identification of MRVI1-interacting proteins by biotin-based proximity labelling reveals NPM–ALK-dependent interaction dynamics

**DOI:** 10.1093/jb/mvaf057

**Published:** 2025-10-24

**Authors:** Kosuke Higashi, Yuuki Tanaka, Hidetaka Kosako, Kazumasa Aoyama

**Affiliations:** Division of Hygienic Chemistry, Faculty of Pharmacy, Keio University, 1-5-30 Shibakoen, Minato-ku, Tokyo 105-8512, Japan; Division of Hygienic Chemistry, Faculty of Pharmacy, Keio University, 1-5-30 Shibakoen, Minato-ku, Tokyo 105-8512, Japan; Division of Cell Signaling, Institute of Advanced Medical Sciences, Tokushima University, 3-18-15 Kuramoto-cho, Tokushima 770-8503, Japan; Division of Hygienic Chemistry, Faculty of Pharmacy, Keio University, 1-5-30 Shibakoen, Minato-ku, Tokyo 105-8512, Japan

**Keywords:** apoptosis, endoplasmic reticulum, MRVI1, NPM–ALK, proteomics, proximity labelling

## Abstract

The murine retrovirus integration site 1 (MRVI1) gene encodes an endoplasmic reticulum (ER)-associated membrane protein involved in calcium signalling, yet its molecular interaction network remains largely undefined. Here, we employed TurboID-based proximity labelling to construct the first comprehensive map of MRVI1-associated proteins in mammalian cells. This analysis identified >700 candidate interactors, including ER-localized factors and components of intracellular trafficking, consistent with the subcellular localization and signalling role of MRVI1. To investigate oncogenic modulation, we examined how co-expression of NPM–ALK—a constitutively active tyrosine kinase implicated in lymphoid malignancies—reshapes the MRVI1 interactome. Quantitative proteomics revealed that while the overall composition of MRVI1-associated proteins was largely preserved, a subset of interactions was selectively enhanced or attenuated by NPM–ALK. The association of MRVI1 with several signalling-related proteins was enhanced by NPM–ALK, including 12 proteins that have all been previously implicated in cancer-related pathways. In contrast, proteins whose interaction with MRVI1 was suppressed were functionally enriched in the Gene Ontology term ‘negative regulation of apoptotic process’. Notably, anti-apoptotic regulators such as DDB1, PHB2 and NOTCH2 showed significantly reduced proximity labelling, suggesting that MRVI1 may participate in apoptosis-related networks disrupted during oncogenic transformation. Together, our findings demonstrate that MRVI1 forms a functionally diverse protein network that can be selectively remodelled by oncogenic signalling. This study not only uncovers potential mechanisms by which MRVI1 contributes to transformation but also provides a valuable proteomic resource for future investigation of MRVI1 function and regulation.

## Graphical Abstract

**Graphical Abstract:**
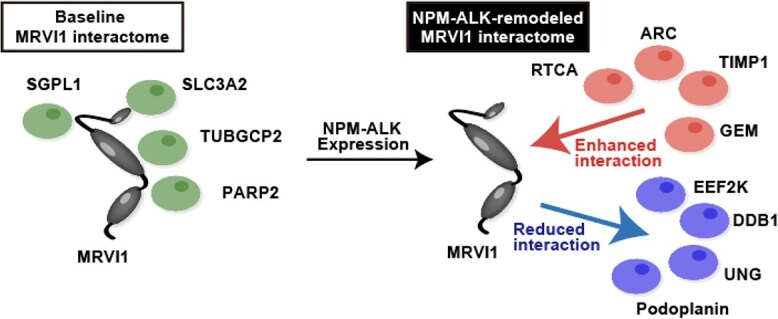

The murine retrovirus integration site 1 (MRVI1) gene was initially identified as a common site of retroviral integration in murine myeloid leukaemia models, suggesting a potential role in haematopoietic regulation and leukaemogenesis *(**1**)*. MRVI1 encodes a Type II membrane protein localized to the endoplasmic reticulum (ER), sharing homology with the lymphoid-restricted membrane protein Jaw1. Its protein product, also referred to as IRAG1, forms a functional complex with the inositol 1,4,5-trisphosphate receptor type 1 (ITPR1) and cGMP-dependent protein kinase Iβ (PKG Iβ), thereby suppressing IP₃-mediated intracellular Ca^2+^ release *(**2**,*  *3**)*. This signalling axis plays a critical role in smooth muscle relaxation, platelet function and cardiovascular homeostasis *(**4**)*.

MRVI1 is widely expressed in various tissues, including smooth muscle, heart, brain and haematopoietic cells, and its functional relevance spans multiple physiological systems *(**5**,*  *6**)*. Notably, MRVI1-deficient mice exhibit altered vascular reactivity and platelet aggregation, highlighting its involvement in NO/cGMP-mediated pathways *(**4**)*. Although MRVI1 is not extensively studied, accumulating evidence suggests its potential role in tumourigenesis. In haematologic malignancies, retroviral insertional mutagenesis of the *MRVI1* locus has been implicated in the disruption of differentiation-related gene networks *(**1**)*. Moreover, *MRVI1* expression is elevated in haematopoietic cells transformed with oncogenic kinases such as BCR-ABL and TEL-PDGFRB *(**4**)*. In solid tumours, *MRVI1* exhibits context-dependent expression: its upregulation correlates with chemoresistance and poor prognosis in ovarian cancer, whereas downregulation via promoter hypermethylation or miRNA-mediated silencing is associated with unfavourable outcomes in endometrial and cervical cancers *(**4**)*.

The NPM–ALK fusion protein, generated by the t(2;5)(p23;q35) chromosomal translocation, is a constitutively active tyrosine kinase that drives oncogenesis in anaplastic large-cell lymphoma (ALCL) *(**7**)*. It activates multiple downstream signalling cascades—including STAT3, PI3K-AKT and ERK pathways—that support malignant proliferation and survival *(**8**,*  *9**)*. In particular, STAT3 has emerged as a key effector of NPM–ALK-driven transcriptional programmes and cellular transformation *(**10**,*  *11**)*. In this context, we previously observed in murine Ba/F3 cells that ectopic expression of NPM–ALK was associated with increased MRVI1 mRNA levels (data not shown), which served as a motivation to investigate the MRVI1 interactome. However, this preliminary finding was not pursued further in the current study. This finding prompted us to investigate the molecular mechanisms by which oncogenic signalling modulates *MRVI1* biology.

Given the limited knowledge surrounding *MRVI1*, we sought to elucidate its protein interaction landscape by proximity-dependent biotin labelling. Furthermore, considering its potential oncogenic modulation, we evaluated how *MRVI1*-associated interactions are influenced by NPM–ALK expression. Through this approach, we aimed to uncover novel regulatory mechanisms and functional implications of *MRVI1* in cancer-related signalling contexts.

## Materials and Methods

### Cells and transfection

Lenti-X 293 T cells (#632180, Clontech) were cultured in DMEM (high glucose, 4.5 g/l Glucose) supplemented with 10% foetal bovine serum and 1% penicillin–streptomycin. Cells were maintained at 37°C in a humidified incubator with 5% CO₂. Transfection was conducted using polyethyleneimine *(**12**)*.

### Plasmids

The CSII-V5-TurboID-IRES-Puro vector was constructed by polymerase chain reaction (PCR) amplification of V5-TurboID from the V5-TurboID-NES-pCDNA3 plasmid (#107169, Addgene) and subcloning it into the CSII-EF-RfA-IRES-Puro backbone (#RDB12869, RIKEN BRC). The PCR was performed using the following primers: forward, 5′-atcgatatcgccaccatgggcaagcccatccccaa-3′ and reverse, 5′-gatgatatcgcggccgcattggatcctgatcctcctcctcctgatcctcctcctccggcagaccgcagact-3′. The CSII-V5-TurboID-MRVI1-IRES-Puro vector was generated by inserting the MRVI1 cDNA obtained by reverse transcription and PCR amplification from mRNA of NPM-ALK-expressing Ba/F3 cells *(**10**)* into the CSII-V5-TurboID-IRES-Puro vector. The PCR was performed using the following primers: forward, 5′-cagggattcccccacattcctgaggatgaggagcc-3′ and reverse, 5′-ctggcggccgcctactgctctgctggcagctcttg-3′. The MSCV-NPM-ALK-IRES-GFP vector previously constructed *(**10**)* was used in this study.

### Antibodies

The following antibodies were used: anti-V5 (#M215–3, Medical & Biological Laboratories; sc-271,944, Santa Cruz), anti-ALK (#3633, Cell Signalling Technology), anti-Tubulin (#MCA77G, Bio-Rad), Anti-Mouse IgG-HRP (#NA931, GE Healthcare; Light Chain Specific, #115–035-174, Jackson), Anti-Rabbit IgG-HRP (#NA934, GE healthcare; Light Chain Specific, #211–032-171, Jackson), Anti-Rat IgG-HRP (#sc-2006, Santa Cruz; #7077, Cell Signalling Technology), and Anti-Rabbit IgG-CoraLite® Plus 594 (Proteintech, Cat. No. RGAR004, 1:1,000) antibodies.

### Biotin-based proximity labelling proteomic analysis

Biotin-based proximity labelling proteomic analysis was performed as described previously *(**13**,*  *14**)* with some modifications, to identify proteins interacting with MRVI1. LentiX-293 T cells transfected with V5-TurboID or V5-TurboID-MRVI1 with or without NPM-ALK were cultured for 2 days in the presence of 500 μM D-Biotin during the last 24 h for induction of protein biotinylation by TurboID. Cells were lysed in guanidine buffer (6 M guanidine-HCl and 100 mM HEPES-NaOH, pH 7.5) containing 10 mM Tris (2-carboxyethyl) phosphine (TCEP) and 40 mM chloroacetamide (CAA). Biotinylated proteins were purified using Tamavidin 2-REV beads *(**13**,*  *14**)* and subjected to liquid chromatography–tandem mass spectrometry (LC–MS/MS) analysis. Data analysis was performed using Proteome discoverer software (Thermo). Proteins significantly enriched in V5-TurboID-MRVI1-expressing cells compared to the control (V5-TurboID alone) were identified based on fold enrichment and statistical significance.

### Gene ontology enrichment analysis

Gene Ontology (GO) enrichment analysis was performed using the Database for Annotation, Visualization, and Integrated Discovery (DAVID) *(**15**,*  *16**)*. Lists of differentially expressed genes (official gene symbols) were uploaded to DAVID, and enrichment analysis was conducted exclusively for the Biological Process (BP) category using the default human background. GO terms with *P*-values <0.05 were considered statistically significant. The result tables were downloaded in tab-delimited format and further processed in Excel software (Microsoft). GO terms were visualized using bar plots generated in Excel software.

### Western blotting

Western blotting was performed as described previously. Cells were washed with PBS twice and lysed in SDS lysis buffer (2% SDS, 20 mM Tris–HCl, pH 8.0) *(**17**)*. The lysates were sonicated, added to the same volume of 2× SDS sample buffer and incubated at 95°C for 10 min. Proteins in lysates were separated by SDS-PAGE, transferred to a PVDF membrane (PALL) *(**18**)*, and reacted with primary and secondary antibodies after blocking with 1% BSA *(**19**)*. For detection of biotinylated proteins, HRP-conjugated streptavidin (#405210, BioLegend) was used. Protein bands were detected with enhanced chemiluminescence reagent (Immobilon Western, Millipore) and ChemiDoc™ Touch imaging system (Bio-Rad) *(**20**)*. The sequential reprobing was performed after the removal of primary and secondary antibodies from membranes in 0.2 M glycine-HCl buffer (pH 2.5) and/or the inactivation of HRP by 0.1% NaN_3_  *(**21**)*. Images were processed for presentation using GIMP software *(**22**)*. Densitometric quantification of western blot signals was performed using ImageJ software *(**23**)*, as described previously *(**24**)*.

### Immunofluorescence microscopy

Immunofluorescence staining was performed essentially as described previously *(**24**)* with minor modifications. Briefly, cells were grown on poly-L-lysine–coated glass coverslips (22 × 22 mm, Matsunami) and transfected as indicated. After incubation with 500 μM biotin for 24 h, cells were fixed in 4% paraformaldehyde for 20 min, washed three times with PBS, and permeabilized in 0.5% Triton X-100/PBS containing 1% BSA for 20 min. Coverslips were incubated with anti-V5 antibody in blocking buffer overnight at 4°C. For the detection of biotinylated proteins, CoraLite® Plus 488-conjugated Streptavidin (#PF00023, Proteintech) was applied in parallel. After washing, Multi-rAb™ CoraLite® Plus 594-Goat Anti-Rabbit Recombinant Secondary Antibody (H + L) (#RGAR004, Proteintech) was applied for 30–60 min at room temperature in the dark. Samples were mounted using Fluoro-KEEPER Antifade Reagent, Non-Hardening Type with DAPI (Nacalai Tesque), which simultaneously stains nuclei. Images were acquired using an FV4000 confocal laser scanning microscope (Evident, Japan).

## Results

### TurboID–MRVI1 expression induces proximity-dependent biotinylation in cells

**Fig. 1:**
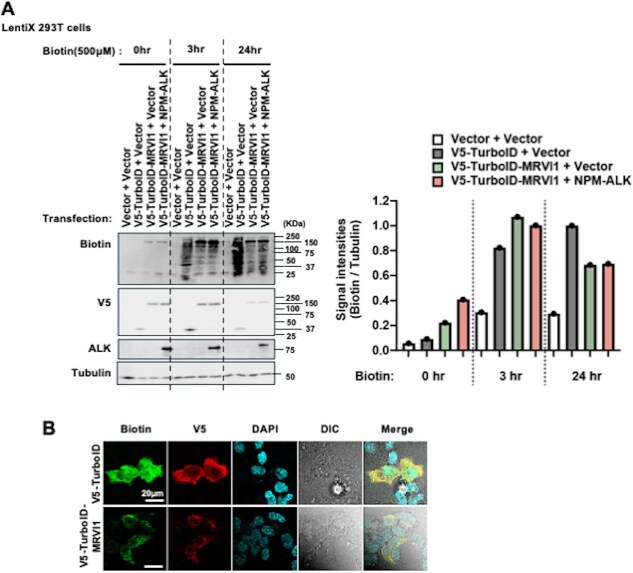
**Induction of protein biotinylation by TurboID-linked MRVI1.** (A) Western blot analysis of Lenti-X 293 T cells transfected with the indicated combinations of MSCV (Empty or NPM–ALK) and CSII (Empty, V5–TurboID or V5–TurboID–MRVI1) vectors. Cells were treated with 500 μM biotin for the indicated time periods (0, 3 or 24 h), and total cell lysates were subjected to immunoblotting using streptavidin-HRP (Biotin), anti-V5, anti-ALK and anti-Tubulin antibodies (left panel). Quantification of biotinylated proteins over time is shown in the right panel, based on signal intensity from the Biotin blot. The levels of biotinylated proteins were normalized to those of Tubulin. A representative result from three independent experiments is presented. (B) Immunofluorescence analysis of Lenti-X 293 T cells expressing V5–TurboID–MRVI1.Cells were stained with anti-V5 (visualized by Alexa Fluor 594), CoraLite® Plus 488-conjugated Streptavidin, and nuclei were counterstained with DAPI included in Fluoro-KEEPER Antifade Reagent.

To examine the protein interaction network of MRVI1 and assess how it is modulated by oncogenic signalling, we employed TurboID-based proximity labelling *(**25**,*  *26**)*. We first constructed a TurboID-tagged MRVI1 (TurboID–MRVI1) expression vector. MRVI1 is a type II ER membrane protein with its N-terminus facing the cytoplasm and its C-terminus oriented towards the ER lumen *(**2**,*  *3**)*. Since our study aimed to investigate cytoplasmic interactions, particularly those related to NPM–ALK signalling, TurboID was fused to the N-terminus of MRVI1. This design enables efficient labelling of cytoplasmic interactors, whereas C-terminal tagging may be useful in future to explore luminal or ER-associated proteins. To validate its expression and enzymatic activity, we performed western blot analysis in LentiX 293 T cells transfected with the indicated vector combinations. As shown in Fig. 1A, V5 immunoblotting confirmed the expression of TurboID–MRVI1. Upon the addition of 500 μM biotin, a marked increase in biotinylated proteins was observed by streptavidin-HRP blotting at both 3 and 24 h after treatment (Fig. 1A and B). Since MRVI1 was transiently expressed, we applied biotin at a relatively high concentration (500 μM) and for a longer duration (24 h) to maximize labelling efficiency. Under these conditions, only minimal morphological changes were observed, and thus this setting was adopted for the present study. Furthermore, immunofluorescence analysis showed that V5–TurboID–MRVI1 was predominantly localized in the cytoplasm (Fig. 1B), which is in line with previous reports *(**2**,*  *3**)* and annotations in the Protein Atlas database *(**27**)* indicating that MRVI1 resides in the endoplasmic reticulum. These results suggest that fusion with MRVI1 altered the predominant localization of the V5–TurboID protein to the cytoplasm, and that TurboID–MRVI1 retained catalytic activity capable of inducing proximity-dependent biotinylation in mammalian cells.

### Proximity labelling identifies MRVI1-interacting proteins

**Fig. 2:**
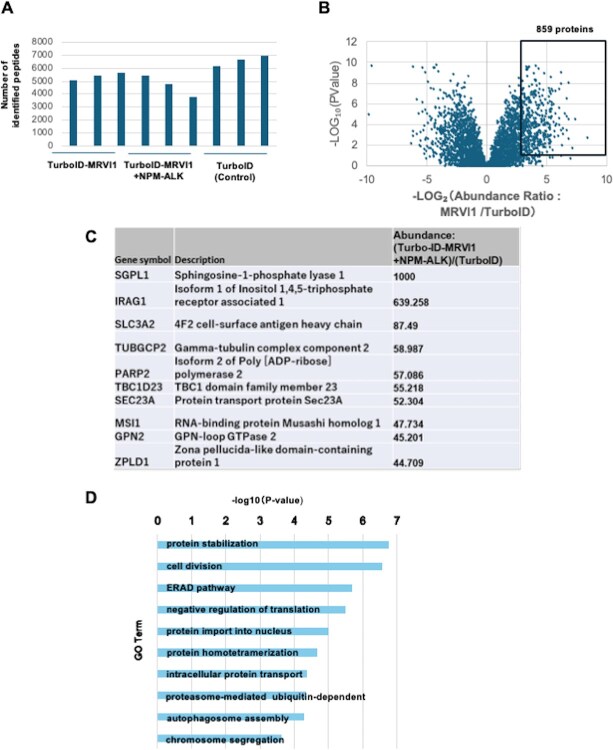
**Identification of MRVI1-interacting proteins by biotin-based proximity labelling.** (A) Bar graph showing the number of peptides identified by proximity labelling by TurboID-MRVI1, with or without co-expression of NPM–ALK, compared to TurboID alone. (B) Volcano plot comparing the abundance of biotinylated proteins between TurboID-MRVI1 and TurboID-alone samples. Proteins significantly enriched in the TurboID-MRVI1 sample (fold change >2 and *P*-value <0.05) are plotted on the right side and highlighted in the black box. These proteins are considered candidate MRVI1-interacting proteins and were subjected to further functional and quantitative analyses in subsequent figures. (C) The list of the top 10 proteins enriched in TurboID-MRVI1 samples based on the abundance. Functional annotations and relative abundance values are included. **(D**) GO enrichment analysis of MRVI1-associated proteins. Top 10 enriched BPs are shown.

Having validated the functionality of the TurboID–MRVI1 construct (Fig. 1), we next performed large-scale proteomic analysis to define its interactome. Lenti-X 293 T cells transfected with TurboID–MRVI1, TurboID–MRVI1 + NPM–ALK or TurboID alone were cultured in the presence of biotin during the last 24 h. After in-solution digestion, biotinylated peptides were affinity-purified and subjected to LC–MS/MS analysis *(**13**)* across three independent biological replicates. The number of identified peptides in each condition is shown in Fig. 2A.

To systematically identify MRVI1-associated proteins, we compared the biotinylated proteome of TurboID–MRVI1 with that of TurboID alone. Volcano plot analysis revealed a broad range of proteins significantly enriched in TurboID–MRVI1-expressing cells (Fig. 2B). These proteins represent high-confidence candidates for proximity-based MRVI1 interactors. To provide an overview of the most robustly enriched candidates, we listed the top 10 proteins ranked by fold change in the MRVI1 samples (Fig. 2C).

In Fig. 2C, IRAG1 was detected as a biotinylated protein in Lenti-X 293 T cells. This signal corresponds to human IRAG1, consistent with the endogenous protein expressed in this cell line. However, since murine MRVI1 and human IRAG1 share highly conserved amino acid sequences, we cannot completely exclude the possibility that mass spectrometry recognized common peptides derived from both species. In addition, IRAG1 may form higher order complexes containing more than one MRVI1 molecule. Under such circumstances, endogenous human IRAG1 molecules incorporated into the same complex could be proximally labelled by TurboID, which may also contribute to the observed biotinylation signal.

To gain broader insight into the functional landscape of the entire MRVI1 interactome, we next performed GO enrichment analysis using all proteins significantly enriched in MRVI1 samples, which are highlighted in black in Fig. 2B. As shown in Fig. 2D, the top enriched biological processes included ‘protein stabilization’, ‘ERAD pathway’, ‘protein import into nucleus’, ‘negative regulation of translation’ and ‘chromosome segregation’. These results suggest that MRVI1 may participate in diverse aspects of protein homeostasis, including endoplasmic reticulum-associated degradation *(**28**,*  *29**)* and regulated intracellular transport. Notably, the enrichment of proteins related to ER-Associated Degradation (ERAD) is consistent with the known subcellular localization of MRVI1 to the endoplasmic reticulum *(**3**)*. These findings that the enrichment of ERAD-related proteins aligns with the known endoplasmic reticulum localization of MRVI1 strongly support the validity of our proximity labelling approach.

### Oncogenic NPM–ALK selectively amplifies MRVI1 interactions

**Fig. 3:**
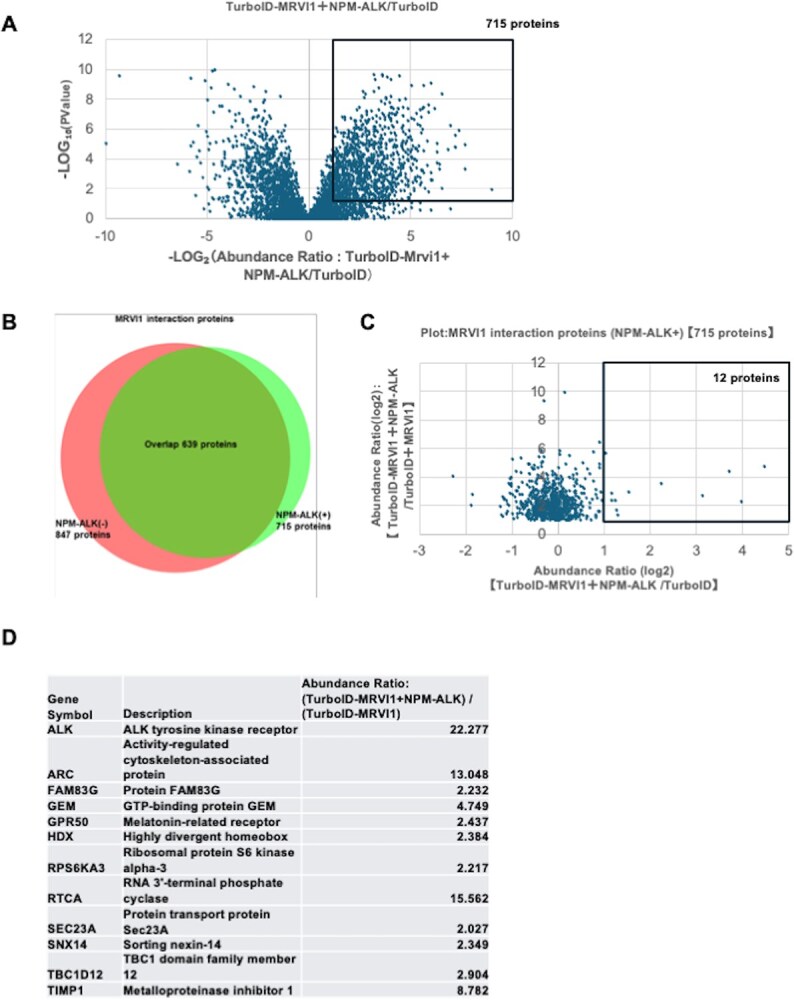
**Identification of proteins showing NPM–ALK-enhanced interaction with MRVI1.** (A) Volcano plot comparing the abundance of biotinylated proteins between TurboID-MRVI1 + NPM–ALK and TurboID-alone samples. Proteins significantly enriched in the presence of NPM–ALK (fold change >2 and *P*-value <0.05) are plotted on the right side and highlighted in the black box. These proteins are considered candidate interactors of MRVI1 under oncogenic signalling conditions. (B) Venn diagram showing the overlap between MRVI1-interacting proteins identified in the absence (left) and presence (right) of NPM–ALK. (C) Scatter plot replotting the data from the boxed region in Fig. 3A (715 MRVI1-interacting proteins identified in the presence of NPM-ALK) comparing the relative abundance (log₂ fold change) of proteins interacting with MRVI1 in the presence versus absence of NPM–ALK. Proteins in the upper-right quadrant—defined by fold change >2 and *P*-value <0.05—represent NPM–ALK-dependent MRVI1 interactors. (D) All 12 MRVI1-interacting proteins showing enhanced abundance upon NPM–ALK co-expression, ranked by fold change. Protein names, gene symbols and functional annotations are shown.

Given that MRVI1 is associated with transformation of haematopoietic cells by oncogenic kinases, including NPM–ALK *(**1**)*, we hypothesized that the protein interaction landscape of MRVI1 may be selectively reprogrammed in the presence of oncogenic signals. To test this, we compared the biotinylated proteomes of TurboID–MRVI1 with and without co-expression of NPM–ALK. Volcano plot analysis revealed numerous proteins specifically enriched in TurboID–MRVI1 + NPM–ALK samples compared to TurboID alone (Fig. 3A). These proteins represent candidate MRVI1 interactors under oncogenic signalling conditions. To further examine the effects of NPM–ALK, we compared MRVI1-associated proteins with and without NPM–ALK expression. As shown in the Venn diagram (Fig. 3B), a large portion of interactors were shared between the two conditions, indicating a conserved core of MRVI1 interactions. Notably, we identified a distinct subset of proteins that showed enhanced association with MRVI1 in the presence of NPM–ALK.

To identify proteins whose interaction with MRVI1 is quantitatively enhanced by NPM–ALK, we directly compared the relative abundance of MRVI1-associated proteins between the two conditions. Scatter plot analysis (Fig. 3C) revealed several proteins with increased proximity labelling upon NPM–ALK expression, and we successfully identified 12 high-confidence candidates that exhibited consistent NPM–ALK–dependent enhancement (Fig. 3D). Notably, all 12 of these candidate proteins have previously been implicated in cancer-related processes, as supported by published studies *(**30–41**)*.

Of note, NPM–ALK itself was among the listed proteins showing increased proximity labelling with MRVI1 (Fig. 3D), consistent with the possibility of a physical interaction. However, given that NPM–ALK is absent from the MRVI1-alone samples, its enrichment reflects the experimental design rather than a comparative increase, and thus was not interpreted as a biologically regulated change.

### A subset of apoptosis-regulating proteins shows reduced interaction with MRVI1 upon NPM–ALK expression

**Fig. 4:**
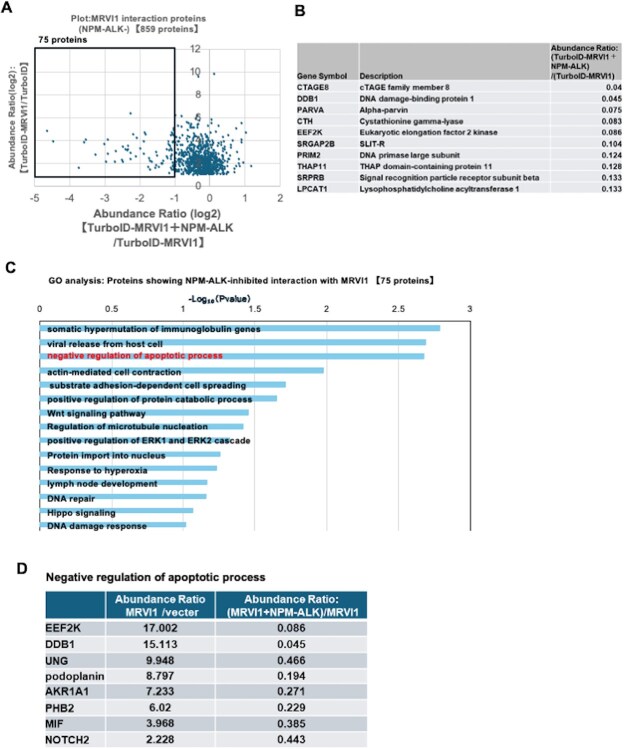
**Identification of proteins showing NPM–ALK-inhibited interaction with MRVI1.** (A) Scatter plot replotting the data from the boxed region in Fig. 2A (859 MRVI1-interacting proteins identified in the absence of NPM–ALK). The *x*-axis shows fold change in MRVI1-alone versus TurboID-alone, and the *y*-axis shows fold change in MRVI1 + NPM–ALK versus MRVI1-alone. Proteins in the lower-right quadrant—defined by fold change <0.5 and *P*-value <0.05—are considered candidates for NPM–ALK-inhibited MRVI1 interaction. (B) Top 10 proteins exhibiting reduced interaction with MRVI1 upon NPM–ALK co-expression. Candidates are ranked based on fold change, derived from comparative proteomic analysis. (C) GO enrichment analysis of proteins showing reduced interaction with MRVI1 upon NPM–ALK expression. (D) Selected proteins from the GO term ‘negative regulation of apoptotic process’ with reduced association to MRVI1 upon NPM–ALK expression. Abundance ratios are shown for MRVI1 versus TurboID (left) and for MRVI1 + NPM–ALK versus MRVI1 (right).

To further define how NPM–ALK influences the MRVI1 interactome, we next focused on proteins whose association with MRVI1 was reduced upon oncogene expression. Comparative analysis between TurboID–MRVI1 and TurboID–MRVI1 + NPM–ALK conditions revealed 75 proteins that were significantly less biotinylated in the presence of NPM–ALK (Fig. 4A). These proteins represent a distinct class of MRVI1 interactors selectively attenuated under oncogenic signalling. The identification of this negatively regulated subset highlights a second, functionally relevant mode of MRVI1 interactome remodelling by NPM–ALK—complementing the enhancement of specific interactions observed in Fig. 3. Representative examples of the top 10 most strongly downregulated proteins are shown in Fig. 4B. To explore the functional characteristics of the NPM–ALK-suppressed interactors, we performed GO enrichment analysis on the 75 downregulated proteins. As shown in Fig. 4C, the results revealed a diverse array of enriched biological processes, including ‘somatic hypermutation of immunoglobulin genes’, ‘viral release from host cell’ and ‘actin-mediated cell contraction’. Among them, ‘negative regulation of apoptotic process’ stood out as a biologically meaningful category, particularly in the context of oncogenesis. Indeed, several proteins annotated with this GO term—such as DDB1 *(**42**)*, PHB2 *(**43**)* and NOTCH2 *(**44**)*—showed markedly reduced association with MRVI1 upon NPM–ALK expression (Fig. 4D). This observation raises the possibility that NPM–ALK may promote malignant transformation, at least in part, by remodelling the MRVI1 interactome in a way that disrupts anti-apoptotic regulatory networks. Together, these results suggest that NPM–ALK orchestrates a selective reorganization of the MRVI1 interactome, potentially contributing to oncogenic signalling and apoptotic resistance.

## Discussion

In this study, we performed TurboID-based proximity labelling to delineate the MRVI1 interactome and investigate how it is remodelled by the oncogenic fusion kinase NPM–ALK. Although MRVI1 has been implicated in haematopoietic regulation and leukaemogenesis through retroviral insertional mutagenesis *(**1**)*, its protein interaction network has remained elusive. Our data provide the first proteomic map of MRVI1-associated factors, uncovering functional modules and regulatory nodes relevant to both physiological and oncogenic signalling contexts.

As shown in Fig. 2D, MRVI1 proximity labelling revealed enrichment of ER-resident proteins and pathways related to ERAD, protein stabilization and intracellular transport. These findings are consistent with the known localization of MRVI1 to the ER and its role in modulating calcium signalling via the IRAG–IP₃R–PKG complex *(**3**,*  *4**)*. ERAD is a conserved quality control mechanism by which misfolded or unassembled proteins in the ER are retrotranslocated into the cytosol, ubiquitinated and subsequently degraded by the 26S proteasome *(**28**,*  *29**,*  *45**,*  *46**)*. This pathway plays a crucial role in maintaining ER homeostasis, particularly under conditions of proteotoxic stress, and is coordinated with the unfolded protein response (UPR) *(**28**,*  *47**,*  *48**)*.

We next examined how MRVI1 interactions are modulated by oncogenic signalling. Upon co-expression of NPM–ALK, a subset of MRVI1 interactions was selectively enhanced (Fig. 3C). Notably, this NPM–ALK-dependent enrichment yielded a set of 12 high-confidence MRVI1-interacting proteins (Fig. 3D), remarkably, all of which have been previously implicated in cancer-related processes *(**30–41**)*. This suggests that NPM–ALK may recruit MRVI1 into a modified signalling network. Since NPM–ALK activates STAT3 and other downstream pathways *(**7**,*  *9**,*  *10**)*, it is plausible that MRVI1 participates in these pathways either directly or via scaffolding functions. Although we initially noted an increase of MRVI1 mRNA in Ba/F3 cells upon NPM–ALK expression, this was a preliminary observation and not reproduced at the protein level in the current Lenti-X 293 T system. We therefore consider this phenomenon unrelated to the main conclusions of the present study.

In addition, although TIMP1 was also enriched in the NPM–ALK condition (Fig. 3D), this is unlikely to reflect direct proximity, as TurboID fused to MRVI1 faces the cytosolic side. We therefore consider that TIMP1 may represent non-specific detection, and our interpretation focuses on the consistent set of NPM–ALK-dependent interactors.

Conversely, a distinct group of proteins exhibited reduced MRVI1 association in the presence of NPM–ALK (Fig. 4A–C). GO analysis of these downregulated interactors revealed enrichment in apoptosis-related terms, especially ‘negative regulation of apoptotic process’. Notable examples include DDB1 *(**42**)*, PHB2 *(**43**)* and NOTCH2 *(**44**)* (Fig. 4D), all of which have established roles in cell survival. These data raise the possibility that NPM–ALK suppresses MRVI1-mediated recruitment of anti-apoptotic regulators, thereby contributing to apoptotic resistance and malignant transformation. Such dual remodelling—gain of pro-oncogenic interactions and loss of apoptotic modulators—highlights the adaptability of the MRVI1 interactome under oncogenic pressure.

Consistent with the ER association of MRVI1, GO analysis in the cellular component category further indicated that the proteins decreased upon NPM–ALK expression were significantly enriched for the ‘endoplasmic reticulum membrane’ (13.5% of proteins, *P* = 0.019). For comparison, analysis of the entire MRVI1-associated proteome at steady state (859 proteins) also demonstrated a strong enrichment for the same category (15.3% of proteins, *P* = 2.57 × 10^−26^), supporting the ER–membrane association of MRVI1 interactors under basal conditions. Together, these results suggest that while MRVI1 interactors are broadly associated with the ER membrane in steady state, NPM–ALK expression selectively attenuates these ER–membrane interactions.

One limitation of this study is the potential cell-type specificity of MRVI1 function. Previous reports have described both tumour-promoting and tumour-suppressive roles of MRVI1, depending on the cellular and disease context *(**1**,*  *3**,*  *4**,*  *49–52**)*. While our proteomic analysis revealed selective remodelling of the MRVI1 interactome by NPM–ALK, we were unable to evaluate how these interactions may differ across diverse cellular backgrounds or transformation states. Nonetheless, our use of a transient transfection-based system in Lenti-X 293 T cells—which are not clonally selected—allowed us to minimize clonal variation and capture a broad, reproducible landscape of MRVI1-associated proteins. This approach enhances the generalizability of our findings and provides a foundational resource for future studies in more specialized or disease-relevant models. Additionally, although changes in proximal proteins could in principle reflect altered expression caused by NPM–ALK, the protein level of MRVI1 itself was unaffected (Fig. 1A), and our comparative design (TurboID–MRVI1 versus TurboID-alone) controls for global expression changes. We therefore interpret the results as reflecting altered interactions with MRVI1.

Taken together, our findings suggest that MRVI1 acts as a dynamic scaffold that integrates signalling and stress-adaptive inputs at the ER membrane. The ability of NPM–ALK to selectively rewire the MRVI1 interactome reveals a previously unrecognized mechanism by which oncogenes may exploit ER-associated networks to promote transformation. Further dissection of MRVI1-associated complexes under defined stress or therapeutic conditions may yield novel insights into its role in cancer biology and may uncover new therapeutic targets.
